# Parity and incident type 2 diabetes in older Chinese women: Guangzhou Biobank Cohort Study

**DOI:** 10.1038/s41598-023-36786-x

**Published:** 2023-06-12

**Authors:** Huimin Su, Chaoqiang Jiang, Weisen Zhang, Feng Zhu, Yali Jin, Karkeung Cheng, Taihing Lam, Lin Xu

**Affiliations:** 1 School of Public Health, Sun Yat-Sen University, 74 Zhongshan 2nd Road, Guangzhou, 510080 Guangdong China; 2 Molecular Epidemiology Research Centre, Guangzhou Twelfth People’s Hospital, Guangzhou, 510620 China; 3 School of Public Health, The University of Hong Kong, Hong Kong, 999077 China; 4 Institute of Applied Health Research, University of Birmingham, Birmingham, B15 2TT UK

**Keywords:** Diabetes, Obesity, Epidemiology

## Abstract

This study examined the association between parity and incident type 2 diabetes in older Chinese women and estimated the mediation effect of adiposity indicators. A total of 11,473 women without diabetes at baseline from 2003 to 2008 were followed up until 2012. We used Cox proportional hazards regression to assess the association between parity and incident type 2 diabetes, and mediation analysis to estimate the mediation effect of adiposity indicators. Compared to women with one parity, the hazard ratio (HR) (95% confidence interval (CI)) for incident type 2 diabetes was 0.85 (0.44–1.63), 1.20 (1.11–1.30), 1.28 (1.16–1.41) and 1.27 (1.14–1.42) for women with parity of 0, 2, 3, and ≥ 4, respectively. The proportion of indirect effect (95% CI) mediated by body mass index, waist circumference, hip circumference, waist-to-hip ratio, waist-to-height ratio and body fat percentage was 26.5% (19.2–52.2%), 54.5% (39.4–108.7%), 25.1% (18.2–49.1%), 35.9% (25.6–74.1%), 50.3% (36.5–98.6%) and 15.1% (− 66.4 to 112.3%), respectively. Compared to women with one parity, women with multiparity (≥ 2) had a higher risk of incident type 2 diabetes and up to half of the association was mediated by abdominal obesity.

## Introduction

According to the International Diabetes Federation, there were 536.6 million people with diabetes worldwide in 2021, with China accounting for about a quarter (140.9 million)^1^. Diabetes and its complications have posed a huge socioeconomic burden in China with no trends of reduction^2^. Pregnancy was known to be associated with dramatic alterations in physiology (e.g., peripheral vasodilatation and increased oxygen demand)^3^, metabolism (e.g., glucose metabolism and lipid metabolism)^4,5^ and lifestyle (e.g., decreased physical activity and increased energy intake)^6,7^, and these changes may have a long-term influence on the health in general and diabetes in particular.

Previous epidemiological studies consistently showed a positive association between parity and risk of type 2 diabetes^8–13^. However, there is no consensus on whether the increased risk for type 2 diabetes is due to childrearing-related issues or metabolic disorders related to childbearing^10,14–16^. Furthermore, the strength of association was generally attenuated after adjusting for obesity indices such as weight, body mass index or waist circumference^11,12,17–21^. But such adjustment could have ignored the effects of obesity in the causal model or pathway. As higher parity was associated with postpartum obesity in later life^22–24^, and the latter especially abdominal obesity can lead to a higher risk of diabetes^25,26^, postpartum obesity could be mediators between parity and incident type 2 diabetes.

Hence, we hypothesized that higher parity was associated with higher risk of type 2 diabetes, and such association was mediated, at least partly, by adiposity with various magnitudes of mediation through different adiposity indicators. We prospectively examined the association between parity and incident type 2 diabetes and quantitatively estimated the magnitude of mediation through adiposity indicators using data from a population-based cohort study in China. Furthermore, to examine whether the association was due to childrearing-related issues rather than metabolic disorders related to childbearing, we also assessed the association between the number of children and incident diabetes was significant in men.

## Results

### Characteristics of participants

GBCS enrolled 30,340 participants from 2003 to 2008, after excluding 8422 men, 8829 not returned for repeated examination and 136 with missing information on parity (n = 82) and type 2 diabetes (n = 54), 13,043 women with all variables of interest were included in the current study. Among them, 1570 participants had type 2 diabetes at baseline and 11,473 women were free of baseline type 2 diabetes. During 43,430 person-years of follow-up (mean = 3.8 years, standard deviation = 1.1 years), 1261 (11.0%) developed incident type 2 diabetes among 11,473 women free of baseline type 2 diabetes. Besides, 4236 men without baseline type 2 diabetes were included for analysis.

Table 1 shows that of the 13,043 women, 270 (2.1%), 3865 (29.6%), 4149 (31.8%), 2547 (19.5%), and 2212 (17.0%) women had parity of 0, 1, 2, 3, ≥ 4, respectively. Compared with nulli- or multi-parity, those with parity one was younger, more educated, had higher household annual income, more non-manual workers, non-smokers, current alcohol users and pre-menopausal women, and had higher family history of diabetes (all P < 0.001). Moreover, nulliparous women had a higher level of physical activity, fewer abortions, fewer OCP users, more history of HRT user, and lower levels of BMI, WC, HC, WHR, WHtR, and body fat percentage (all P < 0.001). Women with multiparity were older and less educated, had more manual workers, more with post-menopausal status, more abortion, less history of HRT user, and higher levels of BMI, WC, HC, WHR, WHtR, and body fat percentage.

**Table 1:** Baseline characteristics by parity in 13,043 women in Guangzhou Biobank Cohort Study.

	Parity					Total	P value
	0	1	2	3	≥ 4		
Number (%)	270 (2.1)	3865 (29.6)	4149 (31.8)	2547 (19.5)	2212 (17.0)	13,043 (100.0)	–
Age, years	59.4 (7.6)	54.9 (4.2)	59.8 (5.2)	63.6 (5.7)	67.0 (5.8)	60.3 (6.7)	< 0.001
Education, N (%)							
Primary or lower	76 (28.2)	536 (13.9)	1,682 (40.6)	1569 (61.6)	1805 (81.6)	5668 (43.5)	< 0.001
Secondary or above	194 (71.9)	3329 (86.1)	2,466 (59.5)	977 (38.4)	407 (18.4)	7373 (56.5)	
Occupation, N (%)							
Manual	151 (56.8)	2174 (56.8)	2,468 (59.8)	1823 (71.7)	1808 (82.0)	8424 (65.0)	< 0.001
Non-manual	68 (25.6)	903 (23.6)	961 (23.3)	409 (16.1)	183 (8.3)	2524 (19.5)	
Other	47 (17.7)	748 (19.6)	696 (16.9)	309 (12.2)	214 (9.7)	2014 (15.5)	
Household annual income, RMB/year (US$1 ~ = RMB 6 Yuan), N (%)							
< 10,000	35 (13.0)	74 (1.9)	118 (2.9)	145 (5.7)	299 (13.5)	671 (5.2)	< 0.001
10,000–	133 (49.4)	1084 (28.1)	1,228 (29.6)	896 (35.2)	751 (34.0)	4092 (31.4)	
≥ 30,000	67 (24.9)	2311 (59.8)	1,788 (43.1)	694 (27.3)	363 (16.4)	5223 (40.1)	
Unknown	34 (12.6)	395 (10.2)	1,012 (24.4)	808 (31.8)	798 (36.1)	3047 (23.4)	
Ever smoking, yes, N (%)	8 (3.0)	28 (0.7)	77 (1.9)	89 (3.5)	158 (7.2)	360 (2.8)	< 0.001
Alcohol use, N (%)							
Never	218 (81.7)	2759 (71.8)	3,298 (80.0)	2065 (81.5)	1802 (82.1)	10,142 (78.2)	< 0.001
Former	8 (3.0)	123 (3.2)	89 (2.2)	57 (2.3)	61 (2.8)	338 (2.6)	
Current	41 (15.4)	963 (25.1)	736 (17.9)	411 (16.2)	333 (15.2)	2484 (19.2)	
Physical activity, N (%)							
Inactive	18 (6.7)	388 (10.0)	326 (7.9)	161 (6.3)	149 (6.7)	1042 (8.0)	< 0.001
Minimally active	102 (37.8)	1401 (36.3)	1645 (39.7)	999 (39.2)	882 (39.9)	5029 (38.6)	
Active	150 (55.6)	2076 (53.7)	2178 (52.5)	1387 (54.5)	1181 (53.4)	6972 (53.5)	
Menopausal status, yes, N (%)	243 (90.3)	3213 (83.1)	3942 (95.1)	2489 (97.8)	2184 (98.9)	12,071 (92.6)	< 0.001
Number of abortions	1.2 (1.2)	1.3 (1.0)	1.5 (1.2)	1.6 (1.4)	1.6 (1.7)	1.5 (1.3)	< 0.001
Oral contraceptive pill use, yes, N (%)	6 (2.3)	622 (16.1)	1007 (24.3)	468 (18.4)	321 (14.6)	2424 (18.6)	< 0.001
History of hormone replacement therapy, yes, N (%)	11 (4.1)	155 (4.0)	100 (2.4)	38 (1.5)	19 (0.9)	323 (2.5)	< 0.001
Body mass index, kg/m^2^	22.8 (3.3)	23.5 (3.2)	23.9 (3.2)	24.1 (3.4)	24.2 (3.5)	23.8 (3.3)	< 0.001
Waist circumference, cm	73.9 (8.5)	74.8 (8.0)	77.2 (8.0)	79.1 (8.5)	80.2 (8.8)	77.3 (8.5)	< 0.001
Hip circumference, cm	89.0 (6.7)	89.9 (6.1)	90.9 (6.3)	91.5 (6.5)	91.3 (6.8)	90.8 (6.4)	< 0.001
Waist-to-hip ratio	0.83 (0.06)	0.83 (0.06)	0.85 (0.06)	0.86 (0.06)	0.88 (0.07)	0.85 (0.07)	< 0.001
Waist-to-height ratio	0.48 (0.06)	0.48 (0.05)	0.50 (0.05)	0.52 (0.06)	0.53 (0.06)	0.50 (0.06)	< 0.001
Body fat percentage, %^a^	30.4 (6.8)	32.4 (6.9)	33.8 (7.6)	34.3 (7.2)	33.7 (7.2)	33.1 (7.2)	< 0.001
Family history of diabetes, yes, N (%)	39 (14.4)	747 (19.3)	597 (14.4)	229 (9.0)	155 (7.0)	1767 (13.6)	< 0.001

Results are means (standard deviation) unless otherwise indicated.

^a^3814 women with data on body fat percentage.

### Parity and incident type 2 diabetes

Table 2 shows that, after adjusting for age, education, occupation, household annual income, ever smoking, alcohol use, physical activity, menopausal status, number of abortions, OCP use, history of HRT and family history of diabetes, women of parity 2, 3 and ≥ 4, versus parity one, had a higher risk of incident type 2 diabetes, with the HR (95% CI) being 1.20 (1.11–1.30), 1.28 (1.16–1.41) and 1.27 (1.14–1.42), respectively. No significant association between nulliparous and incident type 2 diabetes was found (HR 0.85, 95% CI 0.44–1.63). In parous women, each additional live birth was associated with 13% higher risk of incident type 2 diabetes (HR 1.13, 95% CI 1.05–1.22).

**Table 2:** Associations of parity with incident type 2 diabetes and follow-up glycemic indicators.

	Parity					Per one live birth increment^a^
	0	1	2	3	≥ 4	
Hazard ratio (95% confidence interval)						
Incident type 2 diabetes						
Crude model	1.16 (0.74, 1.80)	1.00	1.49 (1.28, 1.73)***	1.77 (1.50, 2.08)***	1.86 (1.57, 2.20)***	1.22 (1.16, 1.29)***
Model 1	0.85 (0.44, 1.63)	1.00	1.20 (1.11, 1.30)***	1.28 (1.16, 1.41)***	1.27 (1.14, 1.42)***	1.13 (1.05, 1.22)***
β (95% confidence interval)						
Fasting glucose at follow-up, mmol/L						
Crude model	0.14 (− 0.05, 0.33)	0.00	0.23 (0.17, 0.30)***	0.36 (0.28, 0.43)***	0.47 (0.39, 0.55)***	0.16 (0.13, 0.18)***
Model 1	0.64 (0.18, 1.09)**	0.00	0.20 (0.12, 0.28)***	0.30 (0.20, 0.39)***	0.39 (0.27, 0.50)***	0.13 (0.09, 0.16)***
Model 2	0.43 (0.07, 0.79)*	0.00	0.06 (0.001, 0.12)*	0.09 (0.02, 0.17)*	0.17 (0.08, 0.26)***	0.05 (0.03, 0.08)***
2-h post-load glucose at follow-up, mmol/L						
Crude model	0.08 (− 0.31, 0.48)	0.00	0.55 (0.41, 0.69)***	0.93 (0.77, 1.09)***	1.05 (0.88, 1.22)***	0.37 (0.31, 0.42)***
Model 1	− 0.42 (− 1.35, 0.52)	0.00	0.32 (0.16, 0.48)***	0.51 (0.16, 0.48)***	0.49 (0.26, 0.73)***	0.18 (0.10, 0.25)***
Model 2	− 0.47 (− 1.30, 0.35)	0.00	0.14 (0.001, 0.28)*	0.21 (0.03, 0.38)*	0.16 (− 0.05, 0.37)	0.06 (− 0.01, 0.13)
Glycosylated hemoglobin A_1C_ at follow-up, % ^b^						
Crude model	− 0.11 (− 0.30, 0.07)	0.00	0.10 (0.03, 0.17)**	0.21 (0.13, 0.29)***	0.25 (0.16, 0.34)***	0.09 (0.06, 0.12)***
Model 1	0.08 (− 0.36, 0.53)	0.00	0.05 (− 0.02, 0.13)	0.13 (0.03, 0.24)*	0.14 (0.01, 0.26)*	0.05 (0.01, 0.09)*
Model 2	− 0.11 (− 0.48, 0.26)	0.00	− 0.01 (− 0.07, 0.05)	0.01 (− 0.08, 0.10)	0.01 (− 0.09, 0.12)	0.01 (− 0.03, 0.04)

Model 1 adjusted for age, education, occupation, household annual income, ever smoking, alcohol use, physical activity, menopausal status, number of abortions, oral contraceptive pill use, history of hormone replacement therapy, and family history of diabetes; Model 2 additionally adjusted for fasting glucose at baseline.

^a^Restricted to parous women.

^b^4508 women with data on glycosylated hemoglobin A_1C_.

*P < 0.05; **P < 0.01; ***P < 0.001.

After adjusting for multiple factors as above, women with parity 0, 2, 3, and ≥ 4, versus parity one, showed higher level of fasting glucose at follow-up by 0.43 (0.07–0.79) mmol/L, 0.06 (0.001–0.12) mmol/L, 0.09 (0.02–0.17) mmol/L and 0.17 (0.08–0.26) mmol/L, respectively. In parous women, each additional live birth was associated with 0.05 (0.03–0.08) mmol/L higher fasting glucose at follow-up. Moreover, women with parity 2 and 3 showed higher 2hPG at follow-up by 0.14 (0.001–0.28) mmol/L and 0.21 (0.03–0.38) mmol/L, respectively. However, we found no association between parity and HbA_1C_ at follow-up. Sensitivity analyses excluding 972 pre-menopausal women showed similar results (Supplementary Table S1).

In sensitivity analyses, greater number of children was consistently associated with higher risk of incident type 2 diabetes in parous women (Supplementary Table S2). However, we found no association between number of children and incident type 2 diabetes in men. Compared to those with one child, the HR (95% CI) for incident type 2 diabetes was 1.06 (0.82–1.37), 0.92 (0.67–1.26) and 0.98 (0.69–1.40) for men with 2, 3 and ≥ 4 children, respectively.

### Mediating effect of adiposity indicators on the association between parity and type 2 diabetes

Table 3 shows that compared to parity one, women with parity 2, 3 and ≥ 4 had higher levels of BMI, WC, HC, WHR, WHtR and body fat percentage after adjustment. In parous women, each additional live birth was associated with higher levels of BMI (0.28 kg/m^2^, 95% CI 0.20–0.35), WC (1.30 cm, 95% CI 1.11–1.49), HC (0.72 cm, 95% CI 0.57–0.87), WHR (0.008, 95% CI 0.006–0.009), WHtR (0.008, 95% CI 0.006–0.008), and body fat percentage (0.61%, 95% CI: 0.28–0.94) at baseline. Sensitivity analyses examining the associations between parity and obesity changes during follow-up showed similar results, but the association between parity and body fat percentage was attenuated and became not significant (Supplementary Table S3).

**Table 3:** Associations between parity and baseline adiposity indicators.

	Parity					Per one live birth increment^a^
	0	1	2	3	≥ 4	
Body mass index, kg/m^2^	− 0.24 (− 1.20, 0.72)	0.00	0.44 (0.28, 0.60)***	0.72 (0.51, 0.92)***	0.81 (0.57, 1.04)***	0.28 (0.20, 0.35)***
Waist circumference, cm	− 0.81 (− 3.22, 1.61)	0.00	1.82 (1.42, 2.23)***	3.09 (2.57, 3.60)***	3.83 (3.23, 4.43)***	1.30 (1.11, 1.49)***
Hip circumference, cm	− 1.08 (− 2.95, 0.79)	0.00	1.22 (0.91, 1.53)***	1.99 (1.60, 2.39)***	2.07 (1.61, 2.53)***	0.72 (0.57, 0.87)***
Waist-to-hip ratio	0.001 (− 0.017, 0.019)	0.00	0.009 (0.006, 0.012)***	0.015 (0.011, 0.019)***	0.023 (0.018, 0.027)***	0.008 (0.006, 0.009)***
Waist-to-height ratio	− 0.001 (− 0.017, 0.015)	0.00	0.010 (0.008, 0.013)***	0.017 (0.014, 0.021)***	0.023 (0.019, 0.026)***	0.008 (0.006, 0.008)***
Body fat percentage, %^b^	− 1.47 (− 4.55, 1.61)	0.00	1.45 (0.81, 2.09)***	1.93 (1.07, 2.78)***	1.39 (0.36, 2.42)**	0.61 (0.28, 0.94)***

Adjusted for age, education, occupation, household annual income, ever smoking, alcohol use, physical activity, menopausal status, number of abortions, oral contraceptive pill use, and history of hormone replacement therapy.

^a^Restricted to parous women.

^b^3814 women with data on body fat percentage.

**P < 0.01; ***P < 0.001.

Table 4 shows that, in 11,236 parous women, the association between parity and incident type 2 diabetes was partially mediated by adiposity indicators, with the proportion (95% CI) of mediation through BMI, WC, HC, WHR, WHtR and body fat percentage being 26.48% (19.25–52.28%), 54.50% (39.43–108.66%), 25.05% (18.18–49.13%), 35.88% (25.65–74.09%), 50.29% (36.48–98.59%) and 15.13% (− 66.40 to 112.29%), respectively. Besides, the direct effect (i.e., all possible causal mechanisms except the one accounted for the mediator) of parity on risk of incident type 2 diabetes was non-significant after controlling for WC, WHtR or body fat percentage, indicating that the total effect (i.e., the sum of the indirect and direct effect) can be totally explained by WC, WHtR or body fat percentage, respectively.

**Table 4:** Baseline adiposity indicators in mediating the association of parity (≥ 1) with incident type 2 diabetes.

Mediators	Indirect effect (ACME) estimate (95% CI)	Direct effect (ADE) estimate (95% CI)	Total effect estimate (95% CI)	Proportion via mediation % (95% CI)
Body mass index, kg/m^2^	0.0031 (0.0021, 0.0041)	0.0083 (0.0026, 0.0132)	0.0114 (0.0059, 0.0159)	26.5 (19.2, 52.3)
Waist circumference, cm	0.0062 (0.0049, 0.0075)	0.0050 (− 0.0011, 0.0102)	0.0112 (0.0057, 0.0157)	54.5 (39.4, 108.7)
Hip circumference, cm	0.0029 (0.0020, 0.0037)	0.0083 (0.0025, 0.0133)	0.0112 (0.0058, 0.0157)	25.1 (18.2, 49.1)
Waist-to-hip ratio	0.0040 (0.0030, 0.0050)	0.0069 (0.0010, 0.0120)	0.0109 (0.0053, 0.0154)	35.9 (25.6, 74.1)
Waist-to-height ratio	0.0058 (0.0045, 0.0071)	0.0055 (− 0.0004, 0.0107)	0.0113 (0.0058, 0.0158)	50.3 (36.5, 98.6)
Body fat percentage^a^	0.0016 (0.0006, 0.0029)	0.0079 (− 0.0034, 0.0164)	0.0095 (− 0.0015, 0.0176)	15.1 (− 66.4, 112.3)

All mediators were standardized using Z-score to facilitate comparison and interpretation. Adjusted for age, education, occupation, household annual income, ever smoking, alcohol use, physical activity, menopausal status, number of abortions, oral contraceptive pill use, history of hormone replacement therapy, and family history of diabetes as appropriate.

*ACME* average causal mediation effect, *ADE* average direct effect, *CI* confidence interval.

^a^3814 women with data on body fat percentage.

## Discussion

In this population-based study of older women, we found that compared to parity one, women with multiparity (≥ 2) had a significantly higher risk of incident type 2 diabetes, and the association was mediated by obesity indicators, of which the proportion of mediation effects through WC-related obesity indicators was as high as 35.9–54.5%. Our study has added to the literature by quantifying the substantial mediation effects through obesity.

Our results were generally consistent with those from studies in China (two cross-sectional studies and one prospective study)^8–10^ and other settings^11–13,27,28^, showing that higher parity was associated with increased risk of type 2 diabetes. Two meta-analyses^27,28^, and a prospective study from Singapore^11^ showed that higher parity was associated with a higher risk of type 2 diabetes than nulliparous women in a linear pattern. Furthermore, a prospective^10^ and a cross-sectional study^9^ in China showed that both nulli- and multi-parity were associated with a higher risk of diabetes compared to parity one. However, another prospective study using data from 10 countries showed a U-shaped association with the incident diabetes, with the nadir at those with parity 2, although similar risk estimate was found in those with parity one^29^. Results of the above studies generally support the greater parity, the higher risk of diabetes. However, there were also discrepancies in the results with and without adjusting for adiposity. For example, some^8–12^, but not all studies^17–19^ showed significant results even after adjusting for BMI or other adiposity indicators. In a prospective study in Japan, a higher parity was associated with an increased risk of type 2 diabetes in a linear pattern before adjusting for BMI (P for trend = 0.029), but the association was substantially attenuated toward the null after adjusting for BMI (P for trend = 0.12), suggesting adiposity might play a major role in the pathway between higher parity and diabetes^17^. Our findings were also consistent with results from previous studies^22–24^ by showing that parity was positively associated with various obesity indicators including BMI, WC, HC, WHR, WHtR and body fat percentage in later life, which could be due to accumulated weight gain, weight redistribution, and weight retention during pregnancy and puerperium^30^. Furthermore, our mediation analyses further quantified the substantial mediation effect and highlighted that the association between parity and incident type 2 diabetes might be explained by adiposity. Specifically, up to 50% of the association was mediated by abdominal obesity, which is considered to better predict diabetes risk than the commonly used BMI since middle-aged and older women may not change much in weight with age but have a significant fat accumulation in the trunk, predisposing them to abdominal obesity^31^.

We also found that compared with women with parity one, those with nulli- or multi-parity had higher fasting glucose, and women with parity 2–3 had higher level of 2hPG at follow-up, which was not completely consistent with previous studies^9,21,32^. For example, a cross-sectional study from China showed that compared with women with one parity, women with 2 or ≥ 3 parities had higher level of 2hPG but not fasting glucose^9^. Another American cross-sectional study showed no association between parity and fasting glucose without adjusting for reproductive factors^21^, which may open to residual confounding, and their small sample size (N = 3211) may limit the power to detect the role of parity on fasting glucose.

Moreover, previous studies suggested that the association between parity and risk of diabetes may be due to biological influences of childbearing or socioeconomic burden of child-rearing, or both^10,14–16^. A prospective study in China showed that the association between parity and diabetes risk was explained by environmental factors related to childrearing (socioeconomic burden or lifestyle) rather than biological effects of childbearing^10^. However, misdiagnosis of diabetes was a major concern in this study. As diabetes can be asymptomatic and undiagnosed for years, diagnosis of incident diabetes based on information obtained through record linkage with the National Health Insurance System might be substantially underestimated. For example, the 7-year cumulative incident rate was 1.7% for men and 2.0% for women in the above study, which were much lower than those reported in previous studies in China^33,34^. The misclassification of the study outcome might bias the results towards null.

It has been reported that pregnancy can have a long-term adverse effects on insulin resistance^4^. Multiparity women are repeatedly exposed to higher anti-insulin hormones including placental lactogen, progesterone and cortisol during pregnancy, promoting pancreatic β-cell proliferation and subsequent β-cell dysfunction, which can lead to a higher risk of diabetes^4,35^. Moreover, the increase in body weight related to physical inactivity, high-calorie diets and decreased insulin sensitivity during pregnancy might also result in a higher risk of diabetes in later life^36,37^. Specifically, the elevation of insulin resistance during pregnancy may affect the ability to store adipose tissue, leading to the deposition of excess lipids in visceral adipose tissue^38^. Concurrently, the placenta elicits the secretion of corticotropin-releasing hormone, which exerts an effect on the hypothalamic–pituitary–adrenal axis, culminating in heightened concentrations of cortisol. This phenomenon is involved in the pathophysiological underpinnings of obesity, with particular emphasis on abdominal adiposity^39^. In our study, results of the mediation analysis supported the pathway through adiposity, and up to 50% of the association was mediated by abdominal obesity, which was consistent with a Mendelian randomization analysis showing that abdominal obesity increased the risk of type 2 diabetes by aggravating insulin resistance^26^.

The strengths of this study included the prospective design, the comprehensive measurement of glycemic (fasting plasma glucose, 2hPG, and HbA_1C_) and adiposity indicators (BMI, WC, HC, WHR, WHtR and body fat percentage), and the adjustment of a wide range of potential confounders. However, our study had some limitations. First, women with very high parity might have diabetes at baseline and were not included in the study. Hence, our HRs could be underestimated. Second, although a wide range of potential confounding factors were adjusted, residual confounding could not be ruled out. For example, adiposity before pregnancy might affect both parity and diabetes, but such information was not available in our study. However, we examined the associations between parity and obesity changes during follow-up, which might to some extent mitigate the confounding effect. Finally, as all participants in GBCS were permanent Guangzhou residents, potential confounding due to cultural differences and genetic background was minimized but the generalizability of the results to other populations may be limited.

## Methods

### Study sample

The Guangzhou Biobank Cohort Study (GBCS) is a population-based cohort study with baseline data collected from September 2003 to January 2008 in 30,430 middle aged or older participants. All surviving participants were invited for the follow-up examination from March 2008 to 2012. Details of GBCS have been reported previously^40^. Briefly, GBCS is a 3-way collaboration among Guangzhou Twelfth People’s Hospital and the Universities of Hong Kong, China, and Birmingham, UK. The Guangzhou Medical Ethics Committee of the Chinese Medical Association approved the study, and all participants provided written informed consent before participation. All procedures were performed in accordance with relevant guidelines and regulations.

### Exposure

Parity refers to the number of biological live births, which was the same as a previous study from GBCS^41^. Parity was classified into five categories, i.e., 0 (nulliparity), 1, 2, 3, and ≥ 4 (grand-multiparity), with parity of 1 as reference.

### Outcomes

The primary outcome was incident type 2 diabetes and the secondary outcomes were glycemic indicators including fasting glucose, two-hour post-load glucose (2hPG) and glycosylated hemoglobin A_1C_ (HbA_1C_) measured at the follow-up examination. Because of constraints in funding, HbA_1C_ was measured in 4508 women only who returned for follow-up examination. Fasting glucose was measured by Shimadzu CL-8000 Clinical Chemistry Analyzer (Shimadzu, Kyoto, Japan) at baseline and follow-up. 2hPG was measured 2 h after 75-g oral glucose administration in all participants except those with self-reported physician-diagnosed diabetes or on anti-diabetic treatment. Type 2 diabetes was defined according to the guidelines of the American Diabetes Association: fasting glucose ≥ 7.0 mmol/l, 2hPG ≥ 11.1 mmol/l, and/or self-reported physician-diagnosed diabetes or anti-diabetic treatment during follow-up^42^.

### Confounders and mediators

Confounders (i.e., factors associated with both parity and incident type 2 diabetes in univariate analysis or reported in the literature) included age, education, occupation, household annual income, ever smoking, alcohol use, physical activity, number of abortions, menopausal status, oral contraceptive pill (OCP) use, history of hormone replacement therapy (HRT) and family history of diabetes. Education was categorized as primary or lower, and secondary or above. Occupation was categorized as manual (agricultural work, factory work, or sales and services), non-manual (administrative/managerial, professional/technical, or military/police), and others (housewife or retired). Household annual income was categorized as < 10,000, 10,000–30,000, ≥ 30,000 RMB/year (US$1 ~  = RMB￥6), and unknown. Alcohol use was categorized as never, former, and current users. Ever smoking (former plus current, as the number of each was small) was dichotomized into yes or no. Physical activity was measured by a validated Chinese version of the International Physical Activity Questionnaire (IPAQ) and categorized as inactive, minimally active and active^43^. Number of abortions included the number of spontaneous abortions and induced abortions. Menopausal status, OCP use, history of HRT and family history of diabetes were dichotomized as yes or no, respectively.

Mediators (i.e., factors lie in the causal pathway between parity and incident type 2 diabetes) included body mass index (BMI), waist circumference (WC), hip circumference (HC), waist-to-hip ratio (WHR), waist-to-height ratio (WHtR), and body fat percentage, which were measured at baseline. The anthropometric measures including weight, height, WC and HC were measured by trained nurses following standard procedures. Using a bioelectrical impedance analyzer (Tanita BF350, Tanita Inc., Japan), body fat percentage was added to the measurements at phase 3 of the baseline, and 3,814 had complete data for analysis. BMI (kg/m^2^) was calculated by weight in kilograms divided by height in meters squared. WHR was calculated by dividing WC (cm) by HC (cm), and the WHtR was calculated by dividing WC (cm) by height (cm).

### Statistical analysis

Chi-square tests were used to compare baseline categorical variables by parity, and one-way analyses of variance (ANOVA) for continuous variables. General linear regression was used to examine the associations of parity with adiposity indicators at baseline and glycemic indicators at follow-up, giving regression coefficient (β) and 95% confidence interval (CI). Generalized estimating equation was used to examine the associations between parity and obesity changes (baseline and follow-up). Cox proportional hazards regression was used to assess the association between parity and risk of incident type 2 diabetes, giving crude and adjusted hazard ratio (HR) and 95% CI. Schoenfeld's residuals were used to assess the proportional hazard assumption and no violation was found (all P > 0.05). To rule out the effect of menopause during follow-up on the outcomes, sensitivity analysis was conducted on baseline postmenopausal women. All participants were followed up from baseline to occurrence of type 2 diabetes or to the date of repeated examination, whichever date came first. For those with newly diagnosed type 2 diabetes at the follow-up examination, the censoring date was defined as the midpoint between the baseline and follow-up examinations.

Mediation analyses were conducted to assess the proportion of the association mediated through each of the adiposity indicators in parous women, including BMI, WC, HC, waist-to-hip ratio (WHR), waist-to-height ratio (WHtR) and body fat percentage at baseline separately. To enable comparison of the effect sizes of the different obesity indicators, each obesity indicator was transformed into Z-score before mediation analysis.

To determine whether the association, if any, was due to biological effects, or due to environmental factors associated with childrearing, we conducted sensitivity analysis on the number of children and incident diabetes in men and women separately. Significant associations between number of children and incident diabetes in men may indicate that environmental factors related to childrearing also played a role in the development of diabetes rather than biological effects related to pregnancy or childbearing in our sample. Data analysis was done using STATA/SE 15.1 with the “mediation” package for the mediation analysis. P values were two-sided, with statistical significance defined by P < 0.05.

## Conclusions

Compared to women with one parity, women with multiparity (≥ 2) had a higher risk of incident type 2 diabetes, and up to 50% of the association was mediated by abdominal obesity. The association was unlikely explained by environmental factors related to childrearing. Our results, if causal, highlight the need for weight management particularly in multiparous women.

## Supplementary Information

Supplementary Information.

## Data Availability

The datasets generated during and/or analysed during the current study are available from the corresponding author on reasonable request.
