# Supplementary material for: Parity and incident type 2 diabetes in older Chinese women: Guangzhou Biobank Cohort Study
Source: Sci Rep. 2023 Jun 12;13:9504. doi: 10.1038/s41598-023-36786-x (PMC10261042; doi:10.1038/s41598-023-36786-x)
Supplement: Supplementary file 1 — Supplementary Information. [file 41598_2023_36786_MOESM1_ESM.pdf]

Title of the manuscript:

Parity and incident type 2 diabetes in older Chinese women: Guangzhou Biobank Cohort Study

Author list:

Huimin Su, MB [a], Chaoqiang Jiang, MD [b], Weisen Zhang, PhD [b,*], Feng Zhu, PhD [b], Yali Jin, MPH [b], Karkeung Cheng, PhD [d], Taihing Lam, MD [b,c], Lin Xu, PhD [a,c,*]

[a] School of Public Health, Sun Yat-sen University, Guangzhou 510080, China

[b] Molecular Epidemiology Research Centre, Guangzhou Twelfth People's Hospital, Guangzhou 510620, China

[c] School of Public Health, the University of Hong Kong, Hong Kong 999077, China

[d] Institute of Applied Health Research, University of Birmingham, Birmingham B15 2TT, UK

* Joint corresponding authors

Corresponding author (1): Professor WS Zhang

Guangzhou Twelfth People's Hospital, Guangzhou 510620, China

Tel: (86) 20-38665762

Email: zwsgzcn@163.com

Corresponding author (2): Professor L Xu

School of Public Health, Sun Yat-sen University,

74 Zhongshan 2nd Road, Guangzhou, Guangdong Province, China

Tel: (86) 20-87335523; Fax: (86) 20-87330446;

Email: xulin27@mail.sysu.edu.cn

Supplementary Table S1. Associations of parity with incident type 2 diabetes and follow-up glycemic indicators after excluding 972 pre-menopausal women

	Parity					Per one live birth increment [a]
	0	1	2	3	≥4	
			Hazard ratio (95% confidence interval)			
**Incident type 2 diabetes**						
Crude model	1.16 (0.74, 1.80)	1.00	1.49 (1.28, 1.73) ***	1.77 (1.50, 2.08) ***	1.86 (1.57, 2.20) ***	1.22 (1.16, 1.29) ***
Model 1	0.85 (0.44, 1.63)	1.00	1.20 (1.11, 1.30) ***	1.28 (1.16, 1.41) ***	1.27 (1.14, 1.42) ***	1.13 (1.05, 1.22) ***
			β (95% confidence interval)			
**Fasting glucose at follow-up, mmol/L**						
Crude model	0.18 (-0.02, 0.38)	0.00	0.24 (0.16, 0.31) ***	0.36 (0.28, 0.44) ***	0.48 (0.40, 0.57) ***	0.16 (0.13, 0.19) ***
Model 1	0.81 (0.33, 1.29) **	0.00	0.20 (0.12, 0.28) ***	0.30 (0.20, 0.40) ***	0.39 (0.28, 0.51) ***	0.13 (0.09, 0.16) ***
Model 2	0.43 (0.07, 0.79) *	0.00	0.06 (0.001, 0.12) *	0.09 (0.02, 0.17) *	0.17 (0.08, 0.26) ***	0.05 (0.02, 0.08) ***
**2-hour post-load glucose at follow-up, mmol/L**						
Crude model	0.14 (-0.29, 0.56)	0.00	0.58 (0.43, 0.73) ***	0.94 (0.77, 1.11) ***	1.06 (0.89, 1.24) ***	0.37 (0.31, 0.42) ***
Model 1	-0.48 (-1.48, 0.52)	0.00	0.35 (0.19, 0.52) ***	0.55 (0.34, 0.76) ***	0.53 (0.29, 0.77) ***	0.18 (0.10, 0.26) ***
Model 2	-0.52 (-1.40, 0.37)	0.00	0.16 (0.02, 0.30) *	0.23 (0.05, 0.42) *	0.18 (-0.03, 0.40)	0.06 (-0.01, 0.13)
**Glycosylated hemoglobin A_{1C} at follow-up, %** [b]						
Crude model	-0.08 (-0.28, 0.12)	0.00	0.10 (0.03, 0.18) **	0.22 (0.13, 0.30) ***	0.26 (0.17, 0.35) ***	0.09 (0.06, 0.12) ***
Model 1	0.19 (-0.29, 0.66)	0.00	0.06 (-0.03, 0.14)	0.14 (0.03, 0.25) *	0.14 (0.01, 0.27) *	0.05 (0.01, 0.09) *
Model 2	-0.02 (-0.41, 0.37)	0.00	-0.01 (-0.08, 0.06)	0.01 (-0.08, 0.10)	0.01 (-0.10, 0.12)	0.004 (-0.03, 0.04)

Model 1 adjusted for age, education, occupation, household annual income, ever smoking, alcohol use, physical activity, number of abortions, oral contraceptive pill use, history of hormone replacement therapy, and family history of diabetes;

Model 2 additionally adjusted for fasting glucose at baseline.

[a] Restricted to parous women.

[b] 4,008 women with data on glycosylated hemoglobin A_{1C}.

*: P<0.05; **: P<0.01; ***: P<0.001.

Supplementary Table S2. Associations of number of children with incident type 2 diabetes in those without baseline type 2 diabetes

	Number of children					Per one child increment [a]
	**0**	**1**	**2**	**3**	**≥4**	
**Men (N=4,236)**						
Crude hazard ratio (95% CI)	0.90 (0.37, 2.20)	1.00	1.27 (0.99, 1.61)	1.23 (0.93, 1.63)	1.40 (1.05, 1.87) *	1.10 (1.01, 1.20) *
Model 1	0.80 (0.32, 1.97)	1.00	1.07 (0.82, 1.38)	0.95 (0.69, 1.31)	1.05 (0.73, 1.49)	1.01 (0.89, 1.12)
Model 2	0.78 (0.31, 1.92)	1.00	1.06 (0.82, 1.37)	0.92 (0.67, 1.26)	0.98 (0.69, 1.40)	0.98 (0.88, 1.10)
**Women (N=11,473)**						
Crude hazard ratio (95% CI)	1.16 (0.74, 1.80)	1.00	1.49 (1.28, 1.73) ***	1.77 (1.50, 2.08) ***	1.86 (1.57, 2.20) ***	1.22 (1.16, 1.29) ***
Model 1	1.08 (0.88, 1.33)	1.00	1.17 (1.09, 1.27) ***	1.24 (1.13, 1.36) ***	1.23 (1.11, 1.37) ***	1.12 (1.04, 1.20) **
Model 2	1.11 (0.90, 1.36)	1.00	1.15 (1.06, 1.24) ***	1.20 (1.10, 1.32) ***	1.19 (1.07, 1.32) **	1.09 (1.01, 1.17) *

Model 1 adjusted for age, education, occupation, household annual income, ever smoking, alcohol use, physical activity, and family history of diabetes; Model 2 additionally adjusted for body mass index.

[a] Restricted to those with children.

*: P<0.05; **: P<0.01; ***: P<0.001.

Supplementary Table S3. Associations between parity and obesity changes during follow-up

	Parity					Per one live birth increment [a]
	0	1	2	3	≥4	
**Body mass index, kg/m^2**	-0.14 (-0.99, 0.72)	0.00	0.59 (0.32, 0.86) ***	0.89 (0.58, 1.20) ***	0.99 (0.67, 1.32) ***	0.33 (0.22, 0.45) ***
**Waist circumference, cm**	-0.66 (-2.90, 1.57)	0.00	1.96 (1.56, 2.36) ***	3.33 (2.81, 3.85) ***	4.15 (3.52, 4.77) ***	1.41 (1.21, 1.61) ***
**Hip circumference, cm**	-0.80 (-2.52, 0.92)	0.00	1.52 (1.11, 1.93) ***	2.87 (1.61, 4.13) ***	3.35 (1.41, 5.30) **	1.21 (0.46, 1.96) **
**Waist-to-hip ratio**	-0.003 (-0.022, 0.017)	0.00	0.007 (0.003, 0.011) **	0.012 (0.006, 0.019) ***	0.019 (0.010, 0.028) ***	0.006 (0.003, 0.009) ***
**Waist-to-height ratio**	-0.001 (-0.014, 0.014)	0.00	0.011 (0.009, 0.014) ***	0.019 (0.016, 0.022) ***	0.024 (0.020, 0.028) ***	0.008 (0.007, 0.010) ***
**Body fat percentage, %** [b]	-0.91 (-3.42, 1.60)	0.00	1.56 (0.09, 3.03) *	2.07 (-0.27, 4.42)	1.92 (-1.20, 5.03)	0.69 (-0.39, 1.78)

Adjusted for age, education, occupation, household annual income, ever smoking, alcohol use, physical activity, menopausal status, number of abortions, oral contraceptive pill use, history of hormone replacement therapy, and the interaction term of parity and visit (i.e., baseline or follow-up).

[a] Restricted to parous women.

[b] 3,814 women with data on body fat percentage.

*: $P<0.05$; **: $P<0.01$; ***: $P<0.001$.
